# A Screen against *Leishmania* Intracellular Amastigotes: Comparison to a Promastigote Screen and Identification of a Host Cell-Specific Hit

**DOI:** 10.1371/journal.pntd.0001253

**Published:** 2011-07-19

**Authors:** Geraldine De Muylder, Kenny K. H. Ang, Steven Chen, Michelle R. Arkin, Juan C. Engel, James H. McKerrow

**Affiliations:** 1 Department of Pathology, Sandler Center for Drug Discovery, University of California San Francisco, San Francisco, California, United States of America; 2 Small Molecule Discovery Center, University of California San Francisco, San Francisco, California, United States of America; New York University School of Medicine, United States of America

## Abstract

The ability to screen compounds in a high-throughput manner is essential in the process of small molecule drug discovery. Critical to the success of screening strategies is the proper design of the assay, often implying a compromise between ease/speed and a biologically relevant setting. Leishmaniasis is a major neglected disease with limited therapeutic options. In order to streamline efforts for the design of productive drug screens against *Leishmania*, we compared the efficiency of two screening methods, one targeting the free living and easily cultured promastigote (insect–infective) stage, the other targeting the clinically relevant but more difficult to culture intra-macrophage amastigote (mammal-infective) stage. Screening of a 909-member library of bioactive compounds against *Leishmania donovani* revealed 59 hits in the promastigote primary screen and 27 in the intracellular amastigote screen, with 26 hits shared by both screens. This suggested that screening against the promastigote stage, although more suitable for automation, fails to identify all active compounds and leads to numerous false positive hits. Of particular interest was the identification of one compound specific to the infective amastigote stage of the parasite. This compound affects intracellular but not axenic parasites, suggesting a host cell-dependent mechanism of action, opening new avenues for anti-leishmanial chemotherapy.

## Author Summary

Leishmaniasis, a disease caused by protozoan parasites of the genus *Leishmania*, is a poverty-related disease threatening 350 million people throughout the world. Drugs currently available to treat this disease are toxic to the patient and drug-resistant parasites are emerging. New therapeutics are therefore needed. Fortunately, interest in confronting the treatment challenges has grown and new technology has led to an increase in high-throughput screens conducted against *Leishmania*. In order to gain insight into the most efficient screening strategy, we compared two methods, one targeting the easily cultured insect-infective promastigote stage of the parasite, and the other, targeting the clinically relevant but more difficult to culture intracellular amastigote stage. We show that while a screen against promastigotes is amenable to automation, it fails to recognize all active compounds. These compounds revealed only by an intracellular assay might act on host cell pathways important for parasite development. Targeting such pathways is an emerging strategy in drug discovery against infectious diseases.

## Introduction

Leishmaniasis is caused by protozoan parasites of the genus *Leishmania*. The disease is endemic in the tropics, subtropics and the Mediterranean basin. There are three main clinical syndromes caused by different species of *Leishmania*. Cutaneous and muccocutaneous leishmaniasis result in large, painful sores that can take many months to heal [1]. Visceral leishmaniasis results in fever, weight loss, and damage to internal organs such as the spleen and the liver and may be fatal if left untreated [2].

*Leishmania* parasites are transmitted to mammalian hosts through the bite of phlebotomine sandflies. The parasites that develop in the mid-gut of the flies, called promastigotes, are flagellated and extracellular. Upon injection in the bloodstream of a mammalian host, promastigotes are rapidly phagocytosed by macrophages where they differentiate into the amastigote form. Amastigotes multiply in the macrophage parasitophorous vacuole, leading to destruction of the host cell and release of free amastigotes into the bloodstream, where they are capable of infecting new phagocytic cells [3].

Current treatment for leishmaniasis relies on chemotherapy, as no efficient vaccine is available. Sodium stibogluconate and amphotericin B have been the first line treatment; however, they have significant side effects and unresponsiveness to sodium stibogluconate has been reported for many years [4]–[6]. A few new anti-leishmanial drugs have been recently released (miltefosine, paromomycin), but they also have drawbacks including cost and toxicity [7]. In addition, it has been shown *in vitro* that in some cases resistance can be easily induced [8].

New therapeutics are therefore urgently needed. Recognition of this need in recent years has led to partnerships between a number of foundations, agencies and universities to support the discovery of anti-parasitic agents, including anti-leishmanials. Lead discovery, one of the bottlenecks in the pipeline for novel anti-leishmanial drugs, would be facilitated by improved high-throughput technology allowing for the ability to screen large number of candidates [9], [10]. Several anti-leishmanial high-throughput screens have been reported [11]–[13]. Primary screens often target the parasite promastigote stage because of ease of culture and manipulation. Indeed, promastigotes from several *Leishmania* species are easily maintained as cell suspension *in vitro*. However, as the promastigote is the form of the parasite in the insect vector, it is not the appropriate target for an anti-leishmanial drug [14]. Culture conditions for axenic amastigotes have been developed in order to facilitate the study of this stage of the parasite [15], [16]. This has allowed amastigotes to be screened in a high-throughput manner [17]. However, expression arrays comparing *L. infantum* axenic amastigotes and amastigotes isolated from macrophages have shown differences in several cellular processes, including metabolism, intracellular transport and response to oxidative stress [18]. These observations highlight the importance of the host macrophage in driving the parasite to specific adaptations. The axenic amastigote model therefore has limitations as it does not encompass many aspects of intracellular parasite development [19]. Compounds active against axenic forms might be unable to reach the intracellular amastigote because of their inability to cross host cell membranes or maintain stability under low pH. Other compounds may need to be metabolized by the macrophage to gain activity. Finally, the macrophage itself might be directly targeted, thereby leading to parasite growth inhibition [20].

We have developed a host cell-based screening assay using a human macrophage cell line infected with *L. donovani*, one of the agents of visceral leishmaniasis. This assay format enables screening of compounds directly against the intracellular stage of the parasite. This assay was used to screen a library of 909 bioactive compounds consisting largely of FDA approved small molecules. In order to compare the efficiency of this screening method with traditional high-throughput screening assays, the same compound library was screened against free living promastigotes. A compound leading to sixty percent parasite growth inhibition at 10 µM was considered a hit in both assays. 59 hits were identified in the promastigote assay of which only 26 were also considered hits in the intracellular amastigote assay. Only one compound was specifically active against the intracellular amastigote stage. We conclude that the promastigote assay fails to identify all active compounds and leads to a rate of 56% false positives.

## Materials and Methods

### Cell culture

THP-1 cells (human acute monocytic leukemia cell line – ATCC TIB202) were grown in RPMI supplemented with 10% Foetal Bovine Serum (FBS) and 50 µM 2-Mercaptoethanol at 37°C in 5% CO2. *L. donovani* promastigotes [strain 1S, clone 2D (MHOM/SD/62/1S-cl2D)] were grown at 27°C in RPMI supplemented with 10% FBS and 10% Brain Heart Tryptose medium (BHT) [21]. Differentiation of promastigotes into axenic amastigotes was achieved by dilution of 5×10^5^ promastigotes in 3 ml of low-pH axenic amastigote media (15 mM KCl; 136 mM KH_2_PO4; 10 mM K_2_HPO4·3H2O; 0.5 mM MgSO4·7H2O; 24 mM NaHCO3; 22 mM glucose; 1 mM glutamine, 1× RPMI 1640 vitamin mix, 10 µM folic acid, 100 µM adenosine, 1× RPMI amino acid mix, 5 µg/ml hemin, 50 U/ml of penicillin, 50 µg/ml of streptomycin, 25 mM MES and 20% FBS. The pH was adjusted to pH 5.66 at 22°C, yielding a final pH of 5.5 at 37°C) [22]. Axenic amastigotes were grown in ventilated flasks at 37°C in 5% CO2.

### Compounds

A library of 909 bioactive compounds was donated by Iconix Biosciences. These compounds were dissolved in DMSO at a stock concentration of 1 mM. Amphotericin B (Sigma) was used as a positive control.

### Promastigote high-throughput assay

*L*. *donovani* promastigotes from an exponentially growing culture were diluted to 10^6^/ml in RPMI containing 10% FBS and 10% BHT. The diluted culture (99 µl/well) was dispensed in sterile 96-well flat white opaque assay plates (Greiner Bio-One) using a WellMate multichannel dispenser (Matrix). 1 µl of 1 mM test compound dissolved in DMSO was added to the plates for a final concentration of 10 µM compound and 1% DMSO. Amphotericin B was added as a positive control (final concentration 2 µM, 1% DMSO) and as a negative control, 1 µl DMSO was added (1% final concentration). Compounds and controls were added to the assay plate with the robotic dispenser Biomek FXp liquid handler (Beckman Coulter). Promastigotes were incubated with the compounds for 72 h at 27°C. The parasites were then lysed by adding 50 µl of CellTiter-Glo (Promega) and placed on an orbital shaker for 5 min at room temperature. After lysis, the resulting ATP-bioluminescence was measured using the Analyst HT plate reader (Molecular Devices). Percentage inhibition of parasite growth was calculated for each well as [1-(RLU_x_-RLU^+^)/(RLU^-^-RLU^+^)]*100 where RLU_x_, RLU^+^ and RLU^-^ are respectively the Relative Light Units for each well, positive (amphotericin B) and negative (DMSO) controls. A screening window coefficient, denoted Z' factor, was used to evaluate the performance of the assay. The Z' factor, calculated as 1-(3σ_c+_+3σ_c−_)/(µ_c+_−µ_c−_) where σ_c+_, σ_c−,_ µ_c+_ and µ_c−_ are respectively the standard deviation and mean values of positive and negative controls, is reflective of the assay signal dynamic range and the data variation associated with signal measurement [23]. For GI_50_ determinations (half maximal inhibitory concentration), compounds were serially diluted 3-fold in DMSO, with final assay concentrations ranging from 50 µM to 0.02 µM (1% final concentration of DMSO). GI_50_ curve fitting was carried out using GraphPad Prism 4 Software (GraphPad Software Inc., San Diego, CA).

### Intracellular amastigote high-throughput assay

Sterile, black, 96-well, clear bottom plates (Greiner Bio-One) were seeded with exponentially growing THP-1 (5×10^5^cells/ml). THP-1 were treated with 0.1 µM phorbol myristate acetate (PMA, Sigma) at 37°C for 48 h to achieve differentiation into adherent, non-dividing macrophages. Maturation of THP-1 cells towards monocyte-macrophage like cells is essential to avoid parasitized cells being overgrown by replicating cells. After activation by PMA, cells were washed and incubated with complete RPMI medium containing stationary phase *L. donovani* promastigotes at a macrophage/promastigote ratio of 1/15. After 4 h incubation at 37°C, non-internalized promastigotes were removed by 2–3 successive washes with RPMI containing 5% FBS and 5% horse serum. Test compounds (10 µM), positive control (2 µM amphotericin B) or negative control (1% DMSO) were then added to the cultures using a Biomek FXp liquid handler (Beckman Coulter). Cultures were incubated at 37°C for 72 h. Cells were then washed with phosphate-buffered saline (PBS), fixed for 30 minutes with 4% formaldehyde, rinsed again with PBS, stained for 2 h with 4′,6′-diamidino-2-phenylindole (DAPI 300 nM) and finally washed with PBS. For GI_50_ determination, compounds were serially diluted 3-fold in DMSO, with final assay concentrations ranging from 50 µM to 0.02 µM (1% final concentration of DMSO). Images were acquired with an INCell Analyzer 1000 automated epi-fluorescent microscope (G.E. Healthcare). The excitation and emission filters used to detect DAPI were 350/50 nm and 460/40 nm respectively. Eight image fields were acquired per well with a 20X objective. The proprietary INCell Developer Toolbox 1.7 software was used for image analysis. Segmentation parameters were set to identify host nuclei with a minimum area of 250 µm^2^ and parasite kinetoplast with an average area of 1 µm^2^. The intensity of parasite nucleus was too low to be detected with a 20X objective. A border, representing the boundary of the cell, was drawn around the nucleus (total area between 700 and 2000 µm^2^). Only parasites found within this area were included in the calculation to eliminate extracellular parasites. False positive parasite detection in the nucleus was also excluded from the calculation. Host cell nuclei and parasite kinetoplasts were counted and the ratio of parasites DNA to host nuclei was selected as the measurement output. Percentage inhibition of parasite growth was calculated as [1-(P/hc_x_-P/hc^+^)/(P/hc^−^-P/hc^+^)]*100 where P/hc_x_, P/hc^+^ and P/hc^−^ are parasite per host cell ratio for every well, positive control (amphotericin B) and negative control (DMSO) respectively. Calculation of Z' factor and GI_50_ curve fitting were carried out as described above.

### Dose response study against axenic amastigotes

*L*. *donovani* axenic amastigotes (5×10^5^ cells/ml in axenic amastigote media) were dispensed in sterile 96-well flat white opaque assay plates (Greiner Bio-One) using a WellMate multichannel dispenser (Matrix). Compounds were serially diluted 3-fold in DMSO, with final assay concentrations ranging from 50 µM to 0.02 µM (1% final concentration of DMSO). 1% DMSO and amphotericin B (2 µM, 1% DMSO final concentration) were added as negative and positive controls respectively. Axenic amastigotes were incubated with the compounds for 72 h at 37°C with 5% CO2. Parasite viability was then measured using CellTiter-Glo as described above. Calculation of Z' factor, percentage of parasite growth inhibition and GI_50_ curve fitting were carried out as described above.

## Results

### Development of an image-based high-throughput assay for drug screening against intracellular *L. donovani*

We developed a 96-well plate, cell-based assay simple to manipulate and reproducible, enabling screening of a high number of compounds against intra-macrophage *L. donovani*.

**Figure 1:**
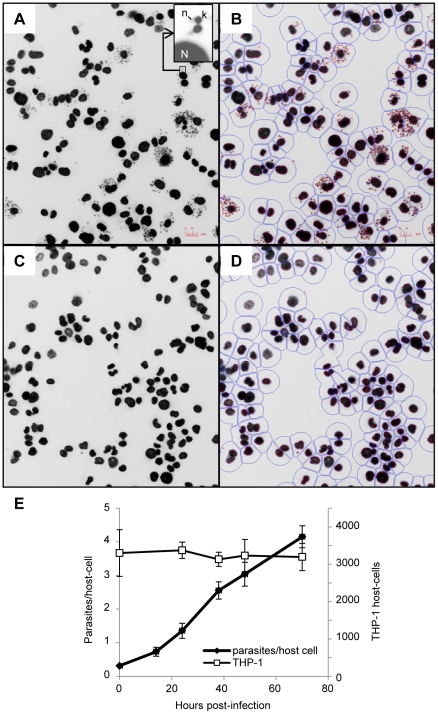
Infection of THP-1 with *L. donovani*: detection, segmentation and growth of host cell and parasite. A–D. Detection and segmentation of THP-1 host cell and *L. donovani* intracellular amastigotes. Images obtained with the INCell Analyzer 1000 (20X) of THP-1 cells infected with *L. donovani* and treated with 1% DMSO (A, B) or 2 µM amphotericin B (C, D). Insert shows the relative fluorescence of DAPI-stained parasite kinetoplast (k) and nucleic DNA (n) and host cell nucleus (N). Segmentation of host cell nuclei and parasite kinetoplast using INCell developer toolbox software (B, D). Red outline: parasite kinetoplast, blue outlines: host cell nucleus and border representing the boundary of the host cell. E. Evolution of the number of parasites and THP-1 host cells in a 72 h time course. THP-1 and *L. donovani* were counted at several time points after infection using the INCell 1000. White squares: average number of host nuclei per well (n = 8); black circles: average number of parasites counted per well divided by the total number of host nuclei per well (n = 8).

**Figure 2:**
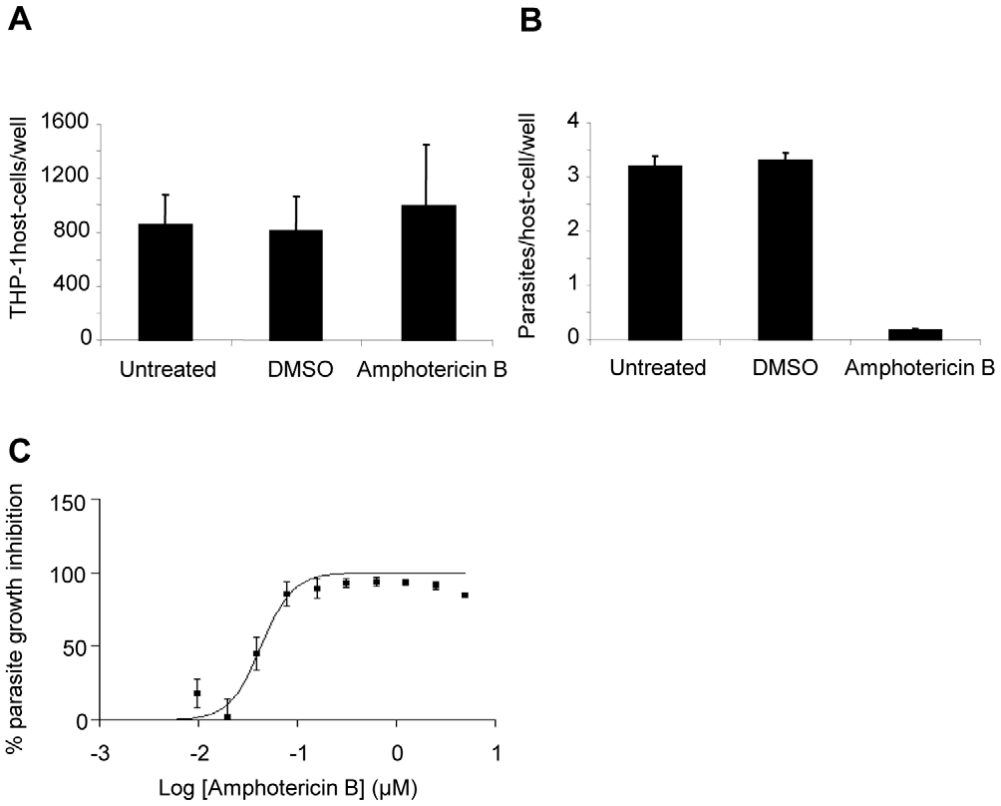
Treatment of infected THP-1 with DMSO and amphotericin B. A. Number of infected THP-1 counted per well treated or not with 1% DMSO or 2 µM amphotericin B. B. Number of parasites counted per well divided by the number of host nuclei per field. C. Dose response curve for amphotericin B plotting the percentage of parasite growth inhibition. Values are mean from at least 3 independent experiments.

The human leukemia monocyte cell line THP-1 has been commonly used as a model for *Leishmania* infection and has been described as a suitable model for drug screening [24], [25]. *In vitro* infection of macrophages by *Leishmania* and analysis of intracellular parasite growth requires a method allowing for robust detection, discrimination and counting of parasites and host cells. In our setting, THP-1 cells infected with *L. donovani* were stained with the DNA marker DAPI (4′,6′-diamidino-2-phenylindole) allowing the visualization of host cell nuclei and parasite kinetoplasts. Images collected with an INCell Analyzer 1000 fluorescent microscope showed a significant size difference between host cell nuclei and parasite kinetoplasts. This feature was exploited for image segmentation and determination of the number of host cells and parasites (Figure 1A–D). The ratio between total number of parasites and total number of host cells was calculated for each well. In addition, counts of host cell nuclei were used as a quantitative measure of cell toxicity induced by the compounds. Incubation of *L. donovani* with THP-1 for 4 hours at a ratio of 15 parasites per host cell led to an average infection of 4.1 +/− 0.32 parasites per host cell after 72 h incubation, with an average of 30 +/− 9 percent of the cells infected and no change in the number of host cells (Figure 1E). Growth of parasite and host cells was not affected by 1% DMSO (Figure 2A–B). Amphotericin B, the first line drug used against leishmaniasis, was used as a positive control. At 2 µM amphotericin B did not affect THP-1 host macrophages (Figure 2A) but significantly inhibited growth of intracellular *L. donovani* (Figure 2B) with an estimated GI_50_ of 0.08 µM (Figure 2C). This is comparable to GI_50_ values from previous reports [11], [20].

### Screening of a bioactive small molecule library against *L. donovani* intracellular amastigotes and free living promastigotes

The intracellular amastigote imaging assay described above was used to screen a library of 909 bioactive small molecules (Iconix library). In the primary screen, compounds were assayed in duplicates at 10 µM. The average Z' value calculated per plate based on the positive and negative controls was 0.63, indicating a satisfactory robustness of the assay. Sixty percent parasite growth inhibition in at least one of the replicates was the cut-off arbitrarily determined for hit selection. This low threshold was purposely selected to evaluate sensitivity of the assay and guarantee identification of all active compounds. In addition, compounds toxic to the host cell, determined as inducing more than 20% reduction in THP-1 numbers, were excluded. A total of 27 compounds met these criteria and were selected for further analysis. This list of active chemicals included previously identified anti-leishmanials such as amphotericin B, pentamidine isothionate and tamoxifen citrate, thus validating the ability of the screen to identify molecules active against *Leishmania*.

The Iconix library was screened in parallel against *L. donovani* free-living promastigotes. Promastigote viability was determined after 72 hours incubation with the compounds, using an ATP-bioluminescence assay previously described for high-throughput screening against *Trypanosoma brucei* [26]. This assay measures luminescence produced by luciferase in presence of cellular ATP; the intensity of light is proportional to the amount of ATP released and correlates with the number of viable parasites (data not shown) [27]. Amphotericin B at 2 µM was used as a positive control and 1% DMSO as a negative control. In the primary screen, compounds were assayed at 10 µM. The assay was robust with an average Z' value of 0.72. Consistent with the image-based assay targeting intracellular *L. donovani*, 60% parasite growth inhibition was the cut-off used for active compound selection. Fifty-nine compounds were selected as hits for further validation.

**Figure 3:**
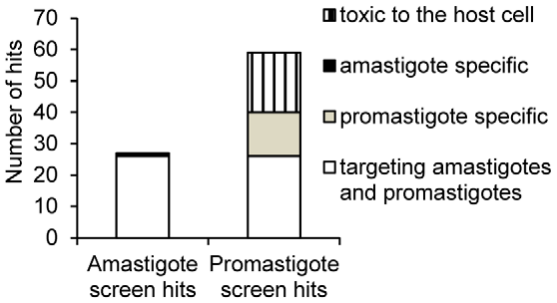
Number of hits identified with the intracellular amastigote and the promastigote primary screens. White bar: number of compounds identified in both screens. Light grey and black bars: number of compounds specifically active against promastigotes and intracellular amastigotes respectively. Hatched bar: number of compounds active against the promastigote stage but determined as toxic against THP-1 host cell in the intracellular amastigote screen.

The comparison of the results obtained for these screens showed that out of the 27 hits identified in the amastigote screen, 26 were also present in the promastigote screen. Only one compound, naloxonazine, showed complete specificity for the intracellular amastigote stage. Out of the 59 compounds identified in the promastigote screen, 19 were considered toxic to the THP-1 macrophage (Figure 3 and Table 1).

**Table 1:** Host cell toxicity and efficacy of compounds against *L. donovani* intracellular amastigotes, promastigotes and axenic amastigotes.

Compounds	GI_50_ THP-1 (µM)	GI_50_ Intracellular amastigote (µM)	GI_50_ Promastigote (µM)	GI_50_ Axenic amastigote (µM)	Selectivity^a^	Specificity^b^
Naloxonazine	33.8	3.49	>50	>50	9.68	14.32
Metergoline	37.14	0.78	15.08	>50	47.22	19.17
R(-)-Apomorphine	>16.5	2.7	38.15	5.20	6.1	14.12
Clotrimazole	>16.5	1.08	12.92	4.56	15.32	11.92
Cetylpyridinium chloride	9.16	0.41	3.30	0.92	22.34	8.05
Aminacrine	12.97	1.9	8.96	>16.5	6.82	4.71
4,4′-Diethylaminoethoxyhexestrol	6.11	1.4	5	10.93	4.36	3.57
Hexachlorophene	16.5	4.54	15.88	14.76	3.62	3.49
Loperamide Hydrochloride	>16.5	5.4	17.3	>50	3.02	3.15
Tamsulosin	27.97	6.3	20	>50	4.43	3.17
Salinomycin	16.09	0.92	2.71	0.06	17.30	2.92
Cycloheximide	21	1.15	2.95	0.39	18.13	2.55
Prazocin Hydrochloride	23.32	6.73	16.74	>50	3.46	2.48
Carvedilol	37.5	6.16	12.41	>16.5	6.07	2.01
Brilliant green	1.64	6	0.10	0.03	15.88	0.02
Antimycin A	>50	2.15	0.1	0.01	500	0.05
BAY 11–7085	>50	>16,5	1.54	0.42	32.46	0.06
Haloprogin	20	6	1.05	0.56	19.04	0.17
Pentamidine Isethionate	>50	9.55	1.69	1.64	29.49	0.17
Zinc Dibutyldithiocarbamate	>50	>50	8.48	>50	5.89	0.17
Amlodipine	>16.5	9.78	3.11	27	5.34	0.31
Nisoldipine	>16,5	32.17	9.85	13.86	1.67	0.30
Parthenolide	>16.5	>50	15.54	4.35	1.06	0.31
Pyrithione zinc	1.043	ND	0.07	0.01	14.9	ND
Thimerosal	12.81	ND	0.49	1.50	26.18	ND
Gramicidin	8.38	ND	0.68	5.76	12.19	ND
Digitonin	13.46	ND	4.376	13.81	3.075	ND
Emetine	1.28	0.082	0.03	1.62	15.58	0.42
Amphotericin B	>50	1.12	1.61	5.42	44.52	1.43
Chlorhexidine	>16,5	1.79	2.81	4.78	9.27	1.56
Oxiconazole	>16,5	6.6	2.65	0.53	2.51	0.40
Bazedoxifene	>16,5	4.8	6.52	>16.5	3.45	1.35
chlorquinaldol	39.3	5.5	6.3	1.72	7.14	1.14
Doxazocin	>16,5	5	8.45	>16.5	3.32	1.69
Aclacynomycine a1	29.1	5.81	9.15	>16.5	5	1.57
Mebeverine	>50	8.5	6.91	2.36	5.88	0.81
Miconazole	>16,5	15.44	7.48	4.73	1.07	0.48
Terfenadine	39.03	14.4	11.92	0.61	2.71	0.82
Tamoxifen Citrate	>16.5	20.79	10.22	>50	1.61	0.49
Auranofin	20.89	21.76	11.11	>50	1.88	0.51
Benzetonium chloride	40.18	10	11.34	10.7	3.54	1.13
Ciclopirox	42	22	15.25	>50	1.90	0.69
Sporidesmin A	0.02	ND	1.18	1.66	0.02	ND
Harringtonin	0.10	4.89	16.61	10.6	0.02	3.39
3,3′,4′,5-tetrachlorosalicylanilide	0.32	ND	1.45	1.38	0.22	ND
Thiram	1.58	10.4	4.62	5.42	0.34	0.44
Idarubicin	1.89	ND	4.65	1.78	0.40	ND
Cerivastatin	7.15	>50	10.8	0.02	0.66	0.22

^*a*^Selectivity is the ratio between parasite GI_50_ and THP-1 GI_50_.

^*b*^Specificity is the ratio between promastigote GI_50_ and intracellular amastigote GI_50_. Specificity value >2 was the cut-off chosen to define a compound as more active against the intracellular amastigote stage; while a specificity value <0.4 indicated a compound more active against promastigotes; compounds with specificity values between 0.4 and 2 were considered active against both stages.

GI_50_ values for these 60 hits (59 identified in the promastigote screen and one intracellular amastigote-specific hit) were then established for both stages of the parasite. 15 compounds (25% of the hits) were equipotent against both stages of the parasite. 14 compounds (23%) were more potent against the intracellular amastigotes while 13 compounds (22%) were more active against the promastigotes. The remaining compounds were toxic to the host cell (Table 1).

As axenic amastigotes have been considered to mimic the intracellular stage of the parasite, we analyzed their sensitivity to the 60 hits described above. This study indicated that compound activity against axenic amastigotes mostly correlated with promastigotes. The specific activity of naloxonazine against intracellular amastigotes was confirmed as this compound showed no activity against promastigotes or axenic amastigotes (Table 1).

### Differential activity of naloxone and naloxonazine, two µ-opioid receptor antagonists, against *L. donovani*

**Figure 4:**
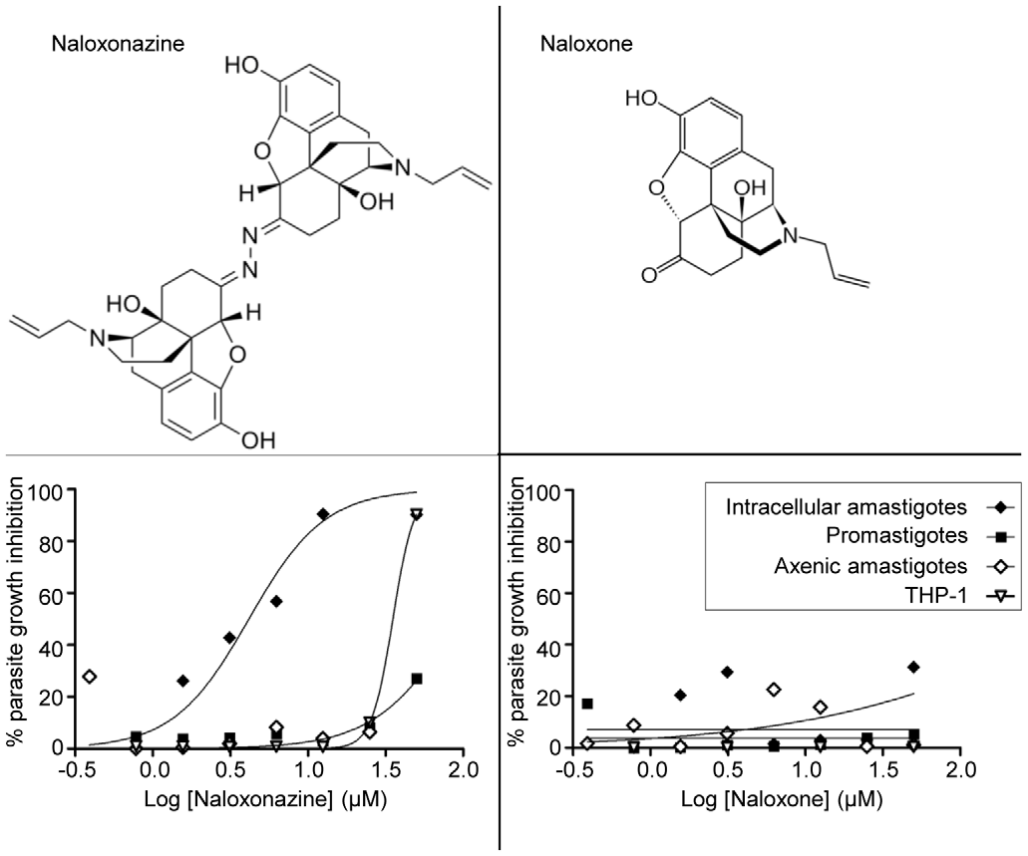
Structure and activity of naloxonazine and naloxone. Lower panels: Dose response curve for naloxonazine (left) and naloxone (right) against intracellular amastigotes (black diamonds), promastigotes (black squares), axenic amastigotes (white diamonds) and THP-1 (white triangles) plotting the percentage of parasite growth inhibition.

The Iconix collection contained two opioid receptor antagonists, naloxone and naloxonazine. The first was not selected as a hit in any of the screens described above while the latter showed specific activity against the intracellular amastigote stage. To confirm these primary observations, the activity of both compounds was tested against promastigotes, intracellular and axenic amastigotes. Naloxonazine exhibited specific activity against intracellular amastigotes (GI_50_ intracellular amastigote: 3.45 µM; GI_50_ THP-1: 33.8 µM; GI_50_ promastigote: >50 µM; GI_50_ axenic amastigote: >50 µM), while naloxone was inactive against all parasite forms and not toxic to the host macrophage (Figure 4). At a curative concentration, the selectivity window of naloxonazine was reduced (GI_90_ intracellular amastigote: 12.5 µM; GI_90_ THP-1: 50 µM), limiting the possibility of using naloxonazine for treatment.

## Discussion

Current chemotherapy for Leishmaniasis has several drawbacks, including cost, toxicities, route of administration, and the emergence of drug resistance. The pipeline for anti-leishmanial drugs therefore needs to be filled with new compounds. As the discovery of new and original leads suitable for optimization and drug development is dependent on the ability to screen many compounds, assays should be rapid, inexpensive and reproducible [14]. In addition, for pathogens displaying several life stages like *Leishmania*, there is a need to determine the best parasite stage to target. In the case of *Leishmania* there are three major options: first, targeting the extracellular living promastigote stage, second the axenic amastigote and third the intracellular amastigote stage. The first and second options meet the reproducibility, rapidity and low cost requirements for high-throughput screens, due to the ease in manipulating promastigotes or axenic amastigotes *in vitro*. This has been demonstrated by Siqueira-Neto *et al*. and Sharlow *et al*. who described screening 26,500 and 200,000 compounds against the *Leishmania* promastigote stage, respectively [11], [12]. These assays monitored parasite viability by measuring products from metabolically active cells (The Alamar Blue assay involves reduction of resazurin into fluorescent resorufin by live cells, while CellTiter-Glo luciferase catalyzes the production of luminescence in the presence of cellular ATP). The axenic amastigotes offer the ability to screen easily the relevant-like stage of the parasite and allow testing the potency of compounds under low pH conditions. However, the major disadvantage of these two approaches is the absence of the host cell in the assays: the natural niche of the parasite is not taken into account and aspects of parasite biology such as host-parasite interactions or accessibility of the target are ignored.

The intracellular amastigote stage has been logically designated as the more relevant target for primary screening against *Leishmania*, but previous methods were labor intensive and would not support automation [19], [28]. Methods traditionally used to detect and estimate the number of intracellular *Leishmania* include Giemsa staining or the use of reporter gene-expressing parasites (green fluorescent protein, luciferase or beta-galactosidase) [29], [30]. Giemsa staining is cumbersome as it needs manual counting. Reporter gene-expressing parasites are a powerful alternative, but stable recombinant parasite populations are required and they do not allow concomitant analysis of the host cell.

Here we describe a high content assay that allows the simultaneous visualization of both host cell nuclei and parasite kinetoplasts by using the DNA marker DAPI. The significant difference in the size of these two organelles facilitates discrimination between host cells and parasites, and thus accurate counting of both entities. Reduction in the number of kinetoplasts gives a measure of inhibition of parasite growth, while a reduction in the number of host cell nuclei is indicative of compound cytotoxicity. Thus, this image-based assay allows the identification of leishmaniocidal as well as leishmaniostatic compounds. All steps of this assay are amenable to automation and could be reduced to 384-well format resulting in a robust high-throughput screening methodology.

To evaluate what differences might be obtained from screening against the extracellular *versus* the intracellular parasite stages, we screened the same set of 909 compounds against both *L. donovani* promastigotes and intracellular amastigotes. We observed that the majority of the hits identified with the intracellular amastigote screen, defined as inducing 60% parasite growth inhibition at 10 µM, were also found in the promastigote screen. One compound showed specific inhibition of intracellular amastigote and was completely inactive against promastigotes. Fifty-six percent of the hits from the promastigote screen were not found in the intracellular amastigote screen. These results indicated that a promastigote screen failed to identify all active compounds and led to 56% of compounds being likely false positives. Thus, while the promastigote stage appears suitable for high-throughput screening, a fraction of the hits would be missed; furthermore, a high rate of false positives is characteristic of primary screens against promastigotes, underlying the importance of evaluating compound activity against intracellular amastigotes at least in a secondary screen. This is in accordance with the findings of Siqueira-Neto *et al.* who found that only 4% of their hits identified in a promastigote primary screen were active in an intracellular context [11]. Advantages of the intracellular amastigote assay include cell-health information, very low cost of consumables and a reduced necessity for secondary assays. The importance of the host cell in the assay was also demonstrated by the dose response study against axenic amastigotes (*i.e.* amastigote-like stage obtained from differentiation of promastigotes *in vitro* in the absence of a host cell); although this parasite form should mimic the intracellular stage, the activity of compounds against axenic amastigotes mostly correlated with promastigotes rather than intracellular amastigotes.

A similar assay was also successfully developed for screening drugs against the intracellular stage of the related parasite *Trypanosoma cruzi* [31]. Screening the Iconix library against intracellular *T. cruzi* identified 56 hits, among which 8 were also hits in the *Leishmania* screen presented here. Six of these were found to be more active against the intracellular amastigote stage of *L. donovani* compared to promastigotes, indicating inter-species activity of compounds only for the intracellular stages of these two different parasites.

Fifty percent of the compounds that were preferentially active against intracellular amastigotes are known to bind mammalian/eukaryotic G protein coupled receptors (opioid receptors, serotonin or dopamine receptors and adrenergic receptors). Heterotrimeric G proteins are absent in trypanosomatids [32] and we could not find convincing homologs of opioid receptors in the *Leishmania* genome. Compounds described as ligands of G protein coupled receptors may have different targets among parasitic proteins, leading to mechanisms of inhibition independent of the host cell. This is the case of a serotonin receptor agonist that interferes with *P*. *falciparum* growth by blocking a surface membrane channel [33], or a κ-opioid agonist active against *T. brucei* whose target remains to be identified as no homolog of κ-opioid receptor is found in the *T. brucei* genome [34]. However, the fact that the ligands of G protein coupled receptors identified in this study, showed a preferential activity towards the intracellular amastigote stage, also highlights the potential value of these host cell signaling pathways as targets. Previous reports demonstrated the involvement of such receptors in inhibition of infection by several intracellular pathogens including *Leishmania* [35]-[37]. Targeting host factors essential for parasite development is an emerging drug discovery paradigm. It is assumed to be less likely to induce drug resistant pathogens and offers the possibility to repurpose drugs by exploiting compounds currently used for diseases unrelated to microbial infection [38], [39].

Interestingly, one compound out of 909 was active against the intracellular amastigote stage but was completely inactive against promastigotes. This compound, naloxonazine, was also inactive against axenic amastigotes, indicating that its activity is dependent on a macrophage function. Naloxonazine is described as an irreversible µ1-opioid receptor antagonist [40]. There is evidence for the presence of opioid receptors on cells of the immune system [41], [42] and it is known that opioids are involved in modulation of host resistance to infectious diseases [43], [44]. The immune response of mice infected with *L. donovani* has been shown to be influenced by the opioid receptor agonist morphine, but the receptors involved and the mechanism leading to this immunomodulation remain unknown [45]. Loperamide, a µ-opioid receptor agonist, was also identified in this study as inhibiting parasite growth, and this compound was also more potent against the intracellular stage of the parasite. Another µ-opioid receptor antagonist, naloxone, also present in the Iconix library, did not show any activity against *L. donovani*. The differential selectivity of naloxone and naloxonazine for opioid receptor binding sites might explain their differential activity against intracellular *L. donovani* [46]. Naloxone is monomer-like while naloxonazine appears as an inverted dimer (Figure 4). The presence of the macrophage appears to be essential for the activity of naloxonazine against *L. donovani*, but the underlying cellular and molecular mechanisms remain to be elucidated. Although naloxonazine itself would not meet the requirement for a therapeutic drug due to the reduced selectivity window at a curative concentration, it would be interesting to analyze other compounds that target the same host cell pathway.

In summary, we report an automated screen against intracellular amastigotes of *L. donovani*. It has the advantage of screening against the relevant stage of the parasite, taking into consideration crucial aspects of its biology, and giving the opportunity to identify host factors critical for the establishment of infection. This is essential for the identification of new, original and diverse lead compounds for anti-leishmanial therapy.
